# Diuretic Treatment in Patients with Heart Failure: Current Evidence and Future Directions—Part II: Combination Therapy

**DOI:** 10.1007/s11897-024-00644-2

**Published:** 2024-02-01

**Authors:** Cuthbert J.J, Cleland J.G.F, Clark A.L

**Affiliations:** 1 Centre for Clinical Sciences, Hull York Medical School, University of Hull, Cottingham Road, Kingston-Upon-Hull, East Yorkshire, UK; 2 Department of Cardiology, Castle Hill Hospital, Hull University Teaching Hospitals Trust, Castle Road, Cottingham, East Yorkshire UK; 3 Robertson Centre for Biostatistics, Glasgow Clinical Trials Unit, University of Glasgow, Glasgow, UK

**Keywords:** Diuretic treatment, Combination therapy, Loop diuretic, Decompensated HF, Acetazolamide, Thiazide, Digoxin, Steroid, Oral salt, Tolvaptan, Sodium-glucose co-transporter 2 inhibitors

## Abstract

**Purpose of Review:**

Fluid retention or congestion is a major cause of symptoms, poor quality of life, and adverse outcome in patients with heart failure (HF). Despite advances in disease-modifying therapy, the mainstay of treatment for congestion—loop diuretics—has remained largely unchanged for 50 years. In these two articles (part I: loop diuretics and part II: combination therapy), we will review the history of diuretic treatment and current trial evidence for different diuretic strategies and explore potential future directions of research.

**Recent Findings:**

We will assess recent trials, including DOSE, TRANSFORM, ADVOR, CLOROTIC, OSPREY-AHF, and PUSH-AHF, and assess how these may influence current practice and future research.

**Summary:**

There are few data on which to base diuretic therapy in clinical practice. The most robust evidence is for high-dose loop diuretic treatment over low-dose treatment for patients admitted to hospital with HF, yet this is not reflected in guidelines. There is an urgent need for more and better research on different diuretic strategies in patients with HF.

## Introduction

Most patients admitted to the hospital with heart failure (HF) exhibit substantial water and salt retention requiring treatment with intravenous (IV) loop diuretics, [1, 2] a therapeutic strategy that has remained largely unchanged for the last 60 years [3]. Although loop diuretics alone may be sufficient for some patients, there is a ceiling of treatment beyond which increasing the dose does not greatly increase diuresis. Resistance to the actions of escalating doses of loop diuretics is common,[4, 5] which can often be overcome by adding a different class of diuretic agent.

There are many possible adjuncts to loop diuretic therapy, including thiazide diuretics, acetazolamide, sodium-glucose co-transporter 2 inhibitors (SGLT2I), arginine vasopressin (AVP) antagonists, hypertonic saline, and oral salt. Each intervention has evidence to support its use as an adjunct to loop diuretic treatment, [6–12].

The European Society of Cardiology HF guidelines recommend the use of thiazide diuretics or acetazolamide in patients who fail to respond adequately to loop diuretic treatment [13]. However, most patients admitted with severe congestion spend more than a week in hospital [14]. Early use of combination therapy may lead to rapid decongestion, [15] which may shorten hospital stay, reducing the risk of nosocomial infection, falls, and physical deconditioning. This, in turn, may lead to improved quality of life (QoL) and better outcomes [16]. Combination diuretic therapy may also allow a reduction in the dose of loop diuretic required to control congestion, reducing side effects of loop diuretics including diuretic resistance. [17] .

On the other hand, more aggressive diuresis may lead to hypotension, renal dysfunction, and electrolyte abnormalities, which may contribute to diuretic resistance, longer hospital stays, worse QoL, and, in the case of HeFREF, may impede initiation or titration of disease-modifying therapies. In the present article, we review possible adjunctive therapies to loop diuretic agents, discuss recent evidence on combination therapy, and highlight some of the gaps in evidence that remain to be addressed.

## Thiazide or Thiazide-Like Diuretics

**Fig. 1:**
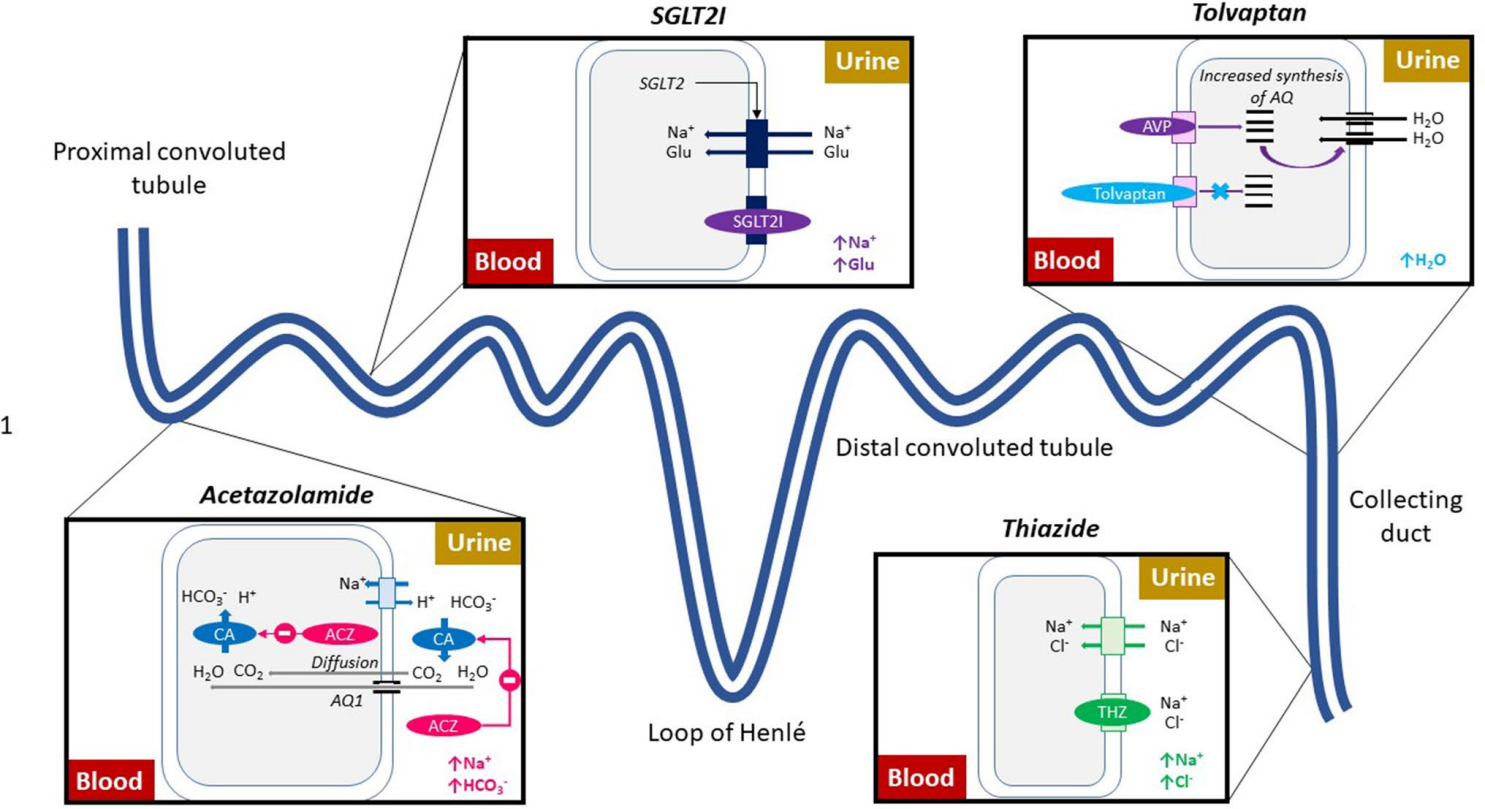
Mechanism of action of different diuretic agents. Abbreviations: CA, carbonic anhydrase; ACZ, acetazolamide; SGLT2I, sodium glucose co-transporter 2 inhibitor; THZ, thiazide; AVP, arginine vasopressin

Thiazide or thiazide-like diuretics (bendroflumethiazide, metolazone, hydrochlorothiazide) inhibit the Na^+^-Cl^−+^ co-transporter in the distal convoluted tubule (DCT) increasing urine sodium excretion which causes a diuresis (Fig. 1). The only evidence to support the use of thiazide and thiazide-like diuretics in patients with HF came from small, randomised trials, often conducted in patients who did not have overt congestion. These generally showed a marked, acute increase in natriuresis and diuresis when given in combination with loop diuretics. [18].

In the CLOROTIC trial, 230 patients admitted to the hospital with HF were randomised to varying doses of oral hydrochlorothiazide or matching placebo based on baseline eGFR in addition to IV furosemide given at usual oral daily dose (mean 80 mg per day) (Table 1)0.7 The co-primary endpoints were changes in body weight and patient-reported symptoms from baseline to 3 days.

**Table 1:** Randomised controlled trials of different adjuncts to diuretic therapy in patients admitted with HF: thiazides and acetazolamide

Trial (date)	Main inclusion criteria	Groups		Patients	Daily dose of IV furosemide	Findings
Thiazide diuretics						
CLOROTIC (2022) [7]	Deemed to need hospitalisation, history of HF, taking LD 80–240 mg per day for ≥ 1 month	Standard care vs. once daily oral HCTZ dosed by renal function Duration: 3 days		*N* = 230 82 years Median eGFR 43 ml/min Median NTproBNP 4720	80 mg in both groups	Greater weight loss in HCTZ arm vs. placebo (− 2.3 kg vs. − 1.5 kg; *P* = 0.002). No difference in patient-assessed SOB Greater urine output in HCTZ vs. placebo during first 24 h (1775 mL vs. 1400 mL; *P* = 0.05) Greater rate of renal dysfunction (46% vs. 17.2%; *P* < 0.001) and hypokalaemia (45% vs. 19%; *P* < 0.001) with HCTZ vs. placebo
		eGFR (ml/min)	Dose			
		> 50	25 mg			
		20–50	50 mg			
		< 20	100 mg			
Acetazolamide						
Imiela and Budaj (2017) [22]	Pulmonary congestion on CxR or signs of congestion O/E; LVEF < 50%	Standard care vs. once daily oral ACZ dosed by body weight Duration: 4 days		*N* = 20 72 years Median creatinine 141 μmol/L Median NTproBNP 8704 ng/L	105 mg in ACZ group 136 mg in SoC group (*P* = NS)	No difference in diuresis and natriuresis (primary endpoints) Greater fluid loss (negative fluid balance) in ACZ arm between days 3–4 due to positive balance in SoC arm
		Weight (kg)	Dose			
		< 75	250 mg			
		75–100	375 mg			
		> 100	500 mg			
DIURESIS-CHF (2019) [23]	≥ 2 clinical signs of congestion; LVEF < 50%; NTproBNP > 1000 ng/L; taking at least 40 mg LD per day prior to admission; at risk of diuretic resistance†	High dose IV LD vs. IV ACZ 500 mg loading followed by 250 mg OD plus low-dose IV LD Duration: 3 days		*N* = 34 80 years Median eGFR 31 ml/min/1.73 m^2^ Median NTproBNP 7849 ng/L	135 mg in ACZ group 240 mg in LD only group By design	No difference in natriuresis after 24 h of treatment (primary endpoint) Diuretic efficacy (natriuresis per mg of bumetanide) greater in ACZ group vs. high dose LD No difference in clinical congestion
ADVOR (2022) [7]	At least one sign of congestion O/E; NTproBNP > 1000 ng/L; taking at least 40 mg LD per day prior to admission	IV ACZ 500 mg OD vs. placebo Duration: 3 days		*N* = 519 78 years Median eGFR 39 ml/min/1.73 m^2^ Median NTproBNP 6173 ng/L	120 mg in each arm	More decongestion in ACZ arm vs. placebo (42% vs. 30%; *P* < 0.001) Shorter admission by 1 day in ACZ arm (9 vs. 10 days) Absolute difference in diuresis on day 2 was 0.5L (4.6 L vs. 4.1 L)

†Defined as as < 1 kg weight loss or < 1 L net fluid loss in the preceding 24 h in patients receiving high dose (> 160 mg per day furosemide equivalents) loop diuretic treatment. Abbreviations: *LD*, loop diuretic; *HCTZ*, hydrochlorothiazide; *eGFR*, estimated glomerular filtration rate; *NTproBNP*, N-terminal pro-B-type natriuretic peptide; *CxR*, chest x-ray; *O/E*, on examination; *LVEF*, left ventricular ejection fraction; *ACZ*, acetazolamide; *SoC*, standard of care; *IV*, intravenous; *OD*, once daily.

Hydrochlorothiazide was associated with greater weight loss (− 2.3 kg vs. − 1.5 kg; *P* = 0.002) but had no effect on symptoms. Although not a pre-specified endpoint, patients randomised to hydrochlorothiazide had fewer signs of congestion after 3 days of treatment. The median length of stay was 7 days and was unaffected by treatment allocation.

There was no difference in the rate of hyponatraemia between hydrochlorothiazide and placebo, but the rate of hypokalaemia (≤ 3.5 mmol/l) was approximately twice as likely with hydrochlorothiazide. There was a trend towards higher rates of all-cause hospitalisation and all-cause mortality at 3 months in the hydrochlorothiazide arm.

The difference in urine output after 24 h was 375 mL greater in the hydrochlorothiazide group (1775 mL vs. 1400 mL; *P* = 0.05). The difference was statistically significant but is not clinically relevant unless the daily difference accumulated throughout the hospital stay, which might have led to a ~ 2.5 L extra diuresis across a 7-day treatment period. However, these data were not collected.

Perhaps the biggest flaw with the CLOROTIC trial is the modest dose of furosemide used as standard care (median 80 mg per day in each arm). This is less than what was used in the low-dose arm of the DOSE trial [19] and a fraction of what was encouraged in the diuretic arm of the CARESS trial [20]. Despite the lack of robust evidence, thiazide diuretics are recommended for patients with HF, but only when given in addition to loop diuretics in those who are diuretic resistant. [14].

### Acetazolamide

Acetazolamide is a carbonic anhydrase (CA) inhibitor. CA catalyses the interconversion between H^+^ and HCO_3_^−^ ions on the one hand to H_2_O and CO_2_ on the other. Inhibition of CA in the lumen of the proximal convoluted tubule (PCT) increases luminal H^+^ concentration, which reduces the activity of the Na^+^-H^+^ exchanger on the apical membrane. Inhibition of intracellular CA reduces the concentration of intracellular H^+^ ions, further reducing the activity of the Na^+^-H^+^ exchanger. The net effect is an increase in urine sodium concentration which may increase diuresis (Fig. 1) 0.2 [21] There have been three randomised controlled trials assessing the effect of acetazolamide on diuresis in patients admitted to hospital with HF (Table 1), of which the ADVOR trial was the largest. [8, 22, 23].

In the ADVOR trial, 519 patients admitted to the hospital with HF, all of whom were already taking loop diuretics prior to admission, were randomised to 500 mg IV acetazolamide or placebo for 3 days. IV furosemide was given at twice the usual daily oral dose. The primary endpoint was the proportion of patients with successful decongestion (no or only trace ankle oedema) after 72 h.

Patients randomised to acetazolamide were more likely to be decongested by day 3 (42% vs. 31%; hazard ratio (HR) 1.47 (95% confidence interval (CI) 1.17–1.82); *P* < 0.001). After 2 days of treatment, acetazolamide was associated with a 0.5 L greater diuresis than placebo (4.6 L (± 1.7 l) vs. 4.1 L (± 1.8 L)). Treatment with acetazolamide also shortened the length of admission by 1 day (9 days (95% CI (9–10 days) vs. 10 days (95% CI 9–11 days) admission duration; *P* < 0.001).

Acetazolamide was well tolerated, and there was no statistical difference in the safety profile compared to placebo. However, there was a trend towards higher rates of renal dysfunction, hypokalaemia, hypotension, and all-cause mortality at 3 months in those who had received acetazolamide.

The mean dose of IV furosemide in the ADVOR trial was 120 mg per day. The effect of acetazolamide on the primary endpoint was driven entirely by those receiving ≤ 120 mg of IV furosemide per day (*N* = 263; HR 1.78 (95% CI 1.33–2.36)). In patients receiving > 120 mg IV furosemide per day, acetazolamide had no effect on the primary endpoint (*N* = 252; HR 1.08 (95% CI 0.76–1.55)).

The use of a clinical composite congestion score as a primary endpoint is problematic because it makes it difficult to be sure what actually improved. Only a third of patients initially had oedema above the knee. Many patients with mild oedema (below the knee) can be managed as an out-patient, particularly if the oral dose of loop diuretic is low, as was the case in ADVOR (median 60 mg per day prior to admission).

The trial was designed prior to the introduction of SGLT2I for the management of HF, but SGLT2i have now become an essential treatment for HF. However, acetazolamide and SGLT2I both increase sodium excretion in the proximal convoluted tubule, and therefore, patients taking SGLT2I were excluded from the ADVOR trial. Whether acetazolamide is effective in the presence of an SGLT2i is uncertain.

### Sodium-Glucose Co-Transporter 2 Inhibitors

The prognostic benefits of sodium-glucose co-transporter 2 inhibitors (SGLT2I) in patients with HeFREF are well established, [24] with modest benefits in HF hospitalisation also seen in patients with HF and a normal LVEF [25]. SGLT2i may exert their benefit by several mechanisms, but diuresis leading to plasma volume contraction and decongestion certainly occurs [26, 27] and might be useful as an adjunct to diuretic therapy.

Large RCTs of patients with relatively stable HF show inconsistent evidence of a diuretic-sparing effect. In patients with HF and a preserved ejection fraction (HeFPEF), randomisation to SGLT2i was associated with a lower rate of initiation of loop diuretic in patients not receiving loop diuretic at randomisation and a lower rate of diuretic intensification, compared to placebo. [28, 29] However, in patients with HF and a reduced ejection fraction (HeFREF), neither the use of loop diuretics nor the mean dose of loop diuretic differed between SGLT2I and placebo groups. [30].

Data from trials of hospitalised with HF are more convincing; the EMPAG-HF, EMPA-RESPONSE-AHF, and EMPULSE (empagliflozin) trials all reported a small, but statistically significant, increase in urine output compared to placebo (Table 2)0.9, [31, 32]

**Table 2:** Randomised controlled trials of different adjuncts to diuretic therapy in patients admitted with HF: SGLT2I and tolvaptan

Trial (date)	Main inclusion criteria	Groups	Patients	Daily dose of IV furosemide	Findings
SGLT2I					
EMPA-RESPONSE-AHF (2018) [8]	NYHA IV and oedema, crackles, or congestion on CxR and raised NTproBNP (≥ 1400 ng/L in SR; ≥ 2000 ng/L in AF) and on IV diuretics	Oral empagliflozin 10mg OD vs. placebo Duration: 4 days	*N* = 79 76 years Median creatinine 115 umol/L Median NTproBNP 4406 ng/L	80 mg in each arm	No difference in patient symptoms, diuretic response, length of admission, or % change in NTproBNP from baseline to day 4 (primary endpoints) Greater urine output with empagliflozin vs. placebo 24 h after randomisation (3442 mL vs. 2400 mL; *P* = 0.01)
EMPAG-HF (2022) [31]	Hospitalisation and NTproBNP > 300 ng/L	Oral empagliflozin 25 mg OD vs. placebo Duration: 5 days	*N* = 59 73 years Median creatinine 98 umol/L Median NTproBNP 4726 ng/L	70 mg in each arm	Greater urine output with empagliflozin after 5 days of treatment 10775 mL vs. 8650 mL (*P* = 0.003) No change in body weight from baseline to day 5
EMPULSE – Diuretic analysis (2022) [32]	NYHA IV and at least two signs of congestion O/E On at least 40 mg IV LD	Oral empagliflozin 10 mg OD vs. placebo Duration: 90 days	*N* = 530 70 years Median eGFR 50 ml/min/1.73 m^2^ Median NTproBNP 3106 ng/L	70 mg in each arm	Greater weight loss (− 3.2 kg vs. − 1.2 kg; *P* < 0.001), reduction in NTproBNP, increase in haematocrit (0.015 vs. − 0.018; *P* < 0.001), and reduction in clinical congestion score (− 1.8 vs. − 1.4; *P* = 0.008) with empagliflozin vs. placebo between baseline and day 15
DAPA-RESIST (2023) [33]	At least one sign of congestion O/E; expected length of stay > 3 days; diuretic resistance†	Oral dapagliflozin 10 mg OD vs. MTZ 5–10 mg OD Duration: 3 days	*N* = 61 79 years Median eGFR 41 ml/min Median NTproBNP 45053 ng/L	255 mg in dapagliflozin arm vs. 185 mg in metolazone arm; *P* = 0.02	No difference in weight loss (− 3.0 kg vs. − 3.6 kg; *P* = 0.11) or change in congestion (measured O/E or on US) between baseline and 4 days
Tolvaptan					
EVEREST (2007) [35] Trials A&B	At least two signs of congestion; known HF; LVEF < 40%;	Oral tolvaptan 30 mg OD vs. placebo Duration: 7 days	*N* = 4133 66 years Mean creatinine 133 µmol/L Median NTproBNP NR	~ 120 mg per day in both arms	Greater weight loss with tolvaptan vs. placebo on day 1 (− 1.7 kg vs. 1.0 kg; *P* < 0.001) and day 7 (− 3.4 kg vs. 2.7 kg; *P* < 0.001). No difference in patient global assessment using a VAS at 7 days More patients noted improvement in breathlessness after day 1 and trend to more patients noting improvement in oedema at day 7
TACTICS (2017) [36]	Breathlessness *or* NT-proBNP > 2000 ng/L and one sign or symptom of congestion; serum sodium < 140 mmol/L	Oral tolvaptan 30 mg OD vs. placebo Duration: 2 days	*N* = 257, 65 years Mean creatinine 129 µmol/L Mean NTproBNP 10,246 ng/L	71 mg in both arms	No effect on symptoms (primary endpoint) but greater weight and fluid loss with tolvaptan vs. placebo during 48 h of treatment. No difference off treatment after 48 h
SECRET of CHF (2017) [9]	NYHA class III or IV symptoms and at least two signs of congestion on examination or CxR Expected to have an “enhanced response” to tolvaptan.†	Oral tolvaptan 30 mg OD vs. placebo Duration: 7 days	*N* = 250 70 years Mean eGFR 47 ml/min/1.73 m^2^ Median BNP 577 ng/L	160 mg in both arms	More patients had “moderately” or “markedly” improved breathlessness at day three with tolvaptan vs. placebo (81% vs. 66%: *P* = 0.02). Greater body weight loss with tolvaptan vs. placebo after 3 days (− 3.5 kg vs. − 2.4 kg; *P* = 0.006). No difference in length of admission or post-discharge outcomes
3T (2020) [37]	At least two signs and symptoms of congestion and loop diuretic resistance.‡	Oral tolvaptan 30 mg OD vs. oral MTZ 5 mg BD vs. IV CTZ 500 mg BD Duration: 2 days	*N* = 60 62 years Mean eGFR 41 ml/min/1.73 m^2^	770 mg vs. 770 mg vs. 675 mg in tolvaptan, MTZ, and CTZ groups, respectively	No difference in weight or fluid loss between the treatments. Each agent caused a diuresis compared to prior to randomisation (*P* = NR)

†Defined as either eGFR < 60 ml/min/1.73 m^2^; serum sodium < 134 mmol/L; or urine output < 125 ml/h in 8 h after first dose of LD. ‡Defined as urine output < 2000 mL in the 12 h before enrolment despite treatment with ≥ 240 mg of IV furosemide. Abbreviations: *NYHA*, New York Heart Association; *eGFR*, estimated glomerular filtration rate; *NTproBNP*, N-terminal pro-B-type natriuretic peptide; *CxR*, chest x-ray; *SR*, sinus rhythm; *AF*, atrial fibrillation; *IV*, intravenous; *O/E*, on examination; *HF*, heart failure; *VAS*, visual analogue scale; *LVEF*, left ventricular ejection fraction; *MTZ*, metolazone; *CTZ*, chlorothiazide; *OD*, once daily; *BD*, bis in dia (twice daily).

The DAPA-RESIST trial (*N* = 61) compared dapagliflozin to metolazone for overcoming diuretic resistance (defined as < 1 kg weight loss or < 1 L net fluid loss in the preceding 24 h despite high dose loop diuretic (≥ 160 mg per day furosemide equivalents)) in patients admitted with HF [33]. Although patients assigned to metolazone received lower concomitant doses of IV furosemide (presumably reflecting less perceived need) and a trend to greater weight loss, the resolution of congestion was similar for each agent. However, metolazone induced more hyponatraemia and a greater increase in urea and creatinine.

In summary, it appears safe to start an SGLT2I in patients who are congested, and the addition of an SGLT2i may enhance a furosemide-induced diuresis, although the effect may be smaller than for metolazone.

### Tolvaptan

Tolvaptan is a selective arginine vasopressin (AVP) V_2_ receptor antagonist. The AVP V_2_ receptor is found on the basolateral membrane of cells in the collecting duct of the renal tubule. Activation of AVP V_2_ increases synthesis of aquaporin-2 channels which increase water reabsorption. Blocking the receptor increases free water excretion [34]. There have been four multi-centre RCTs of arginine vasopressin (AVP) antagonists in patients admitted with HF: EVEREST, [35] TACTICS[36], SECRETs of CHF [10], and the 3T trial. [37].

In the EVEREST trial, patients admitted to hospital with HF were randomised to either tolvaptan 30 mg per day or placebo. There are two aspects to the trial: one focussing on diuretic- and symptom-related endpoints after 7 days of treatment; the other assessing the effect of tolvaptan on long-term outcomes. There was greater weight loss and improvement in breathlessness and peripheral oedema with tolvaptan compared to placebo in the first 7 days but no effect on patient-reported global symptom assessment [38]. Patients assigned to tolvaptan were discharged on lower doses of loop diuretics. However, tolvaptan causes thirst which may have led to a substantial discontinuation rate. In the long term, there was no reduction in cardiovascular hospitalisations or mortality.

In the TACTICS trial, patients admitted with HF were randomised to either tolvaptan 30 mg per day for two days or matching placebo in addition to a fixed dose of IV furosemide (mean dose 71 mg per day). The primary endpoint was the proportion of patients achieving a “moderate” improvement in patient-reported breathlessness at 8 and 24 h after starting treatment. Tolvaptan had no impact on the primary endpoint compared to placebo but was associated with greater weight loss (− 2.8 kg vs. − 1.6 kg; *P* = 0.004) and water loss (− 1948 mL vs. − 1419 mL; *P* = 0.01) in the first 48 h. Differences between the two groups were lost after tolvaptan was stopped. [37].

In the SECRETs of the CHF trial, patients admitted to the hospital with HF who either had renal impairment (eGFR < 60 ml/min/1.73 m^2^), hyponatraemia (≤ 134 mmol/L), or diuretic resistance were randomised to either tolvaptan 30 mg/day vs. matching placebo for 7 days in addition to IV furosemide. The primary endpoint was an improvement in patient-assessed breathlessness after 24 h. As with EVEREST and TACTICS, tolvaptan had no effect on symptoms but was associated with greater weight loss compared to treatment with furosemide alone after 3 days. [10].

The 3T trial was a three-way comparison between tolvaptan, IV chlorothiazide, and oral metolazone in patients admitted with HF who had diuretic resistance defined as urine output < 2.0L in the 12 h before enrolment despite receiving ≥ 240 mg of IV furosemide. Patients were randomised in a 1:1:1 ratio to either tolvaptan 30 mg OD, metolazone 5 mg OD, or chlorothiazide 500 mg twice daily (BD) for 48 h. High doses of IV furosemide were used: 100 mg bolus followed by an infusion of 20 to 30 mg per hour. The primary endpoint was change in weight from baseline to 48 h, and secondary endpoints included urine output and change in patient-reported congestion. [38].

The use of different time periods to measure urine output, and increased doses of loop diuretic, make estimating the diuretic effect of each intervention in the 3T trial difficult. At 48 h, urine output was 7780 mL, 8770 mL, and 9790 mL in the metolazone, chlorothiazide, and tolvaptan arms, respectively, compared to 1170 mL, 1372 mL, and 1022, respectively, in the 12 h prior to randomisation. Cumulative loop diuretic dose was 770 mg, 675 mg, and 770 mg per day, respectively, in the metolazone, chlorothiazide, and tolvaptan compared to 680 mg, 611 mg, and 546 mg per day prior to randomisation.

If urine output was consistent over the 12 h before and 48 h after randomisation, then the greatest increase in daily urine output was in the tolvaptan arm (2044 mL in 24 h prior to randomisation vs. 4895 mL in 24 h after randomisation). However, patients in the tolvaptan arm also had the largest increase in daily loop diuretic dose (546 mg per day before randomisation to 770 mg per day after randomisation. [38].

Tolvaptan may be a useful adjunct to diuretic therapy in patients with diuretic resistance but has no more of a diuretic effect than either IV or oral thiazide diuretics in that circumstance. At present, they are only “suggested” for the treatment of resistant hyponatraemia in the context of congestion. However, the effect of tolvaptan in patients with congestion and hyponatraemia can only be estimated from sub-group analysis of the EVEREST or SECRET of CHF trials—a definitive trial has not been done.

### Digoxin

Digoxin is an antagonist of the Na^+^-K^+^-ATPase pump which is found on the membrane of all human cells. It removes intracellular Na^+^ ions in exchange for K^+^ ions. Na^+^-K^+^-ATPase is found on renal tubular cells throughout the nephron [39]. Inhibition of renal Na^+^-K^+^-ATPase reduces sodium reabsorption, thus reducing renin secretion via tubuloglomerular feedback, [40, 41] which may have natriuretic and diuretic effects in patients with HF. Other cardiotonic steroids, such as ouabain, increase natriuresis and diuresis in animal models. [42].

RCTs of digoxin withdrawal in patients with stable HF conducted more than 20 years ago, long before beta-blockers, MRA, ARNI, or SGLT2i became established, suggested that digoxin might increase systolic blood pressure (~ 5 mmHg) and LVEF (~ 4%), reduce heart rate (~ 10 bpm) and weight (~ 1 kg), and improve renal function. [43–45] Subsequently, a large, long-term RCT found that digoxin reduced heart failure-related hospitalisations and deaths but increased the rate of sudden death, leaving overall mortality unaffected.

However, reductions in HF-related and all-cause hospitalisations appeared substantial for patients with more advanced diseases [46]. Altogether, these data suggest that digoxin could have a role in enhancing diuresis and treating congestion. MRA (by preventing hypokalaemia) and beta-blockers might reduce the risk of sudden death, rendering digoxin safer and more effective in the modern era. Alternatively, digoxin might add little to contemporary treatments for HF. Whether digoxin can enhance a furosemide-induced diuresis for patients receiving contemporary therapy for HF is untested.

### Steroid

**Fig. 2:**
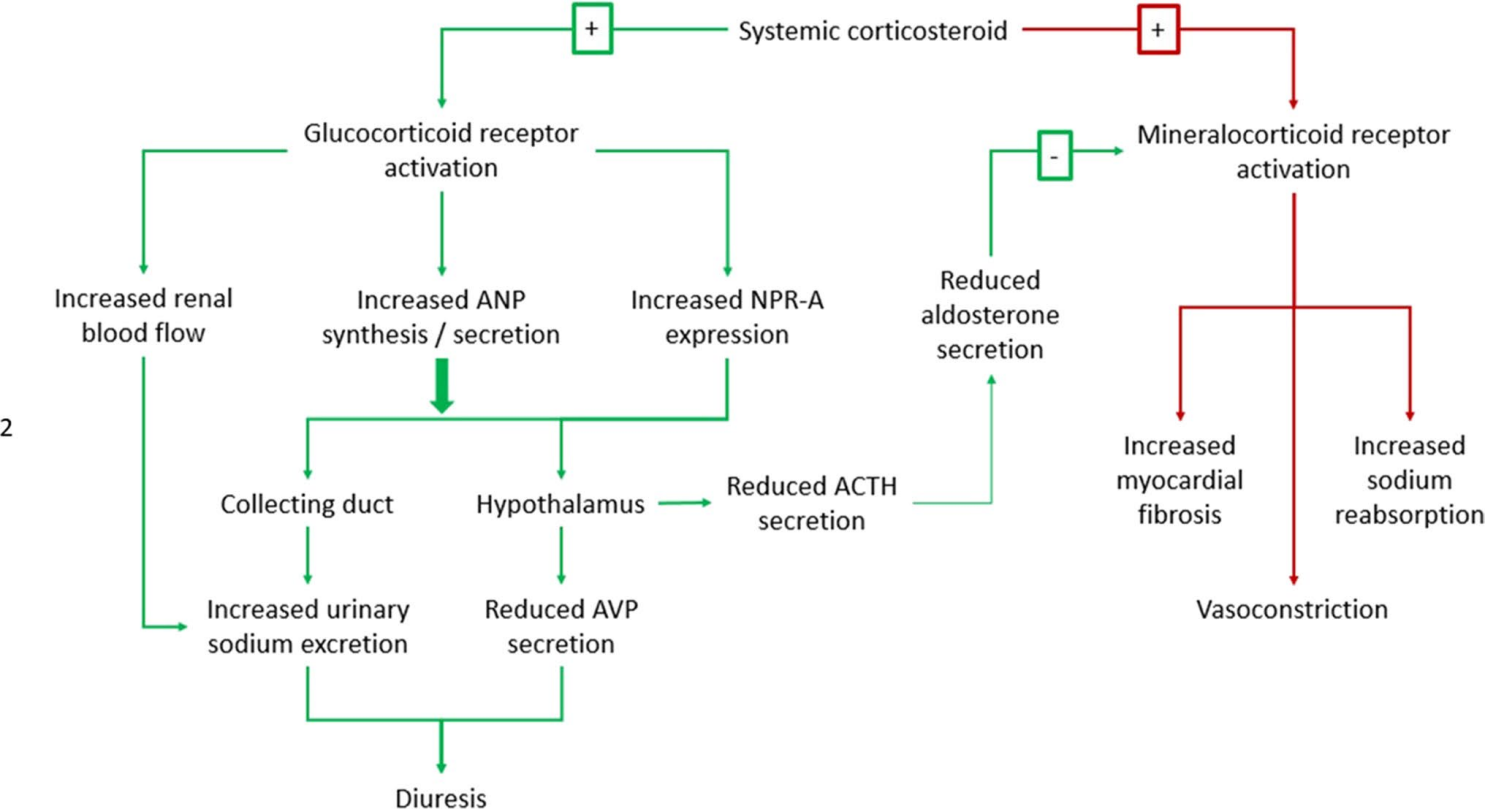
Conflicting and competing mechanisms of corticosteroid benefits and harm in patients with HF. ANP, atrial natriuretic peptide; NPR-A, natriuretic peptide receptor-A; AVP, arginine vasopressin; ACTH, adrenocorticotropic hormone

Prolonged steroid use or high endogenous steroid production is associated with hypertension and an increased risk of cardiovascular disease [47]. Consequently, systemic corticosteroids are considered unsafe in patients with HF [48]. However, several studies suggest that systemic steroids can increase diuresis via activation of glucocorticoid receptors (GR) leading to increases in atrial natriuretic peptide secretion (ANP) and renal blood flow (Fig. 2):

- In a cross-over trial of patients with Addison’s disease (*N* = 7), administration of dexamethasone increased circulating ANP concentration and increased diuresis and sodium excretion compared to glucocorticoid withdrawal. [49]
- In animal studies, activation of GR increases secretion of ANP [50] and expression of natriuretic peptide receptors (NPR-A) in the distal part of the collecting duct (the inner medullary collecting duct (IMCD)), [51] the pulmonary artery [52], and hypothalamus [53]. Activation of NPR-A
  o in the IMCD increases urinary sodium and water excretion; [54]
  o in the pulmonary artery causes vasodilation;
  o in the hypothalamus reduces secretion of AVP[55], and adrenocorticotrophic hormone secretion (which may reduce aldosterone synthesis)0.5 [56]
- In animal studies, activation of GR causes renal vasodilation [57] and increases renal blood flow, [58] via increased nitric oxide and prostaglandin synthesis. [59, 60] The mechanism appears independent of the action of angiotensin II [60] and is limited to the renal vasculature (i.e., not in mesenteric, iliac, or coronary arteries)0.6 [61]
- In animal studies, activation of GR also increases renal dopamine excretion in addition to increased renal blood flow and increased sodium excretion. [62]

In patients with HF, observational data suggest that the addition of steroids to high-dose IV diuretics increases diuresis, [63–65] and in RCTs, steroids are associated with increased diuresis, improved renal function, and, possibly, improved outcome in hospitalised patients with HF [66, 67] and in ambulatory out-patients (Table 3)0.6 [68].

**Table 3:** Observational and trial data of steroids in patients with HF

Trial (date)	Main inclusion criteria	Design, groups, and duration	Patients	Daily dose of IV furosemide	Findings
Liu et al. (2007) [64]	In-patients with NYHA IV, at least two signs of congestion, and diuretic resistance despite sequential nephron blockade.†	Observational Prednisolone 1 mg/kg/day (max 60 mg/day) OD Duration: 28 days	*N* = 13 50 years Mean eGFR 63 ml/min/1.73 m^2^ B/L renal function and NTproBNP not reported	212 mg per day	Steroid induced a diuresis in all patients up to maximum daily urine volume of 7000 mL. Symptoms improved in 12 patients. Mean body weight loss was − 9.4 kg after 28 days. Furosemide dose reduced to a range 20–60 mg per day after 4 days of treatment
Zhang et al. (2008) [65]	In-patients with at least two signs or symptoms of congestion with persistent oedema despite 1 week of IV therapy	Observational Prednisolone 1 mg/kg/day (max 60 mg/day) OD Duration: 9 days	*N* = 35 52 years Mean eGFR 63 ml/min/1.73 m^2^ B/L NTproBNP not reported	Not reported	Mean daily urine volume increased from 1400 mL at B/L to 2400 mL after 9 days. Mean weight loss − 3.2 kg. Mean fasting glucose amongst patients with diabetes (11%) increased during treatment (9.6 mmol/L vs. 12.6 mmol/L; *P* < 0.001). Mean eGFR improved from 63 ml/min/1.73 m^2^ at B/L to 74 ml/min/1.73 m^2^ on day 9
Liu et al. (2006) [66]	Pulmonary oedema on CxR or raised JVP	RCT Prednisolone 1 mg/kg/day (max 60 mg/day) OD vs. placebo Duration: 7 days	*N* = 20 45 years Mean creatinine 99 µmol/L	28 mg per day in steroid group vs. 25 mg per day in pacebo group‡	Greater mean daily diuresis (810 mL) and natriuresis (123 mmol) with prednisolone vs. placebo (*P* < 0.05)
COPE-ADHF (2013) [67]	Orthopnoea, raised JVP, or abdominal pain due to congestion	RCT 20 mg IV dexamethasone loading followed by Prednisolone 1 mg/kg/day (max 60 mg/day) OD vs. SoC Duration: 30 days	*N* = 102 58 years Mean creatinine 104 µmol/L	Not reported	Urine output ~ 500 mL greater with steroid by day 1 increasing to ~ 2500 mL greater by day 7 (*P* < 0.001) Greater weight reduction with steroid vs. SoC (4 kg vs. 2.3 kg; P not quoted) Lower mortality rate at 30 days and 36 months with steroid vs. SoC
PUSH-PATH (2013) [68]	Ambulatory CHF; hyperuricaemia	RCT Prednisolone 1 mg/kg//day (max 60 mg per day) vs. allopurinol 300 mg OD Duration: 28 days	*N* = 34 50 years Mean eGFR 73 ml/min/1.73 m^2^ Mean NTproBNP 6455 ng/L	Not reported	Greater urine output with prednisolone vs. allopurinol by day 10 (3507 mL vs. 1981 mL; *P* < 0.001)

†Defined as failure to achieve negative fluid balance despite treatment with digoxin, furosemide (> 200 mg per day), HCTZ 50 mg per day, spironolactone 50 mg per day, and positive inotropes for at least 3 days. ‡Only 70% of patients receiving loop diuretic therapy. Abbreviations: *NYHA*, New York Heart Association; *eGFR*, estimated glomerular filtration rate; *NTproBNP*, N-terminal pro-B-type natriuretic peptide; *CxR*, chest x-ray; *IV*, intravenous; *O/E*, on examination; *HF*, heart failure; *RCT*, randomised controlled trial; *OD*, once daily.

Oral steroids are not currently recommended for the treatment of HF, although they may be used for co-morbid conditions such as an exacerbation of COPD. Prednisolone and dexamethasone are widely used, but dexamethasone may be more appropriate for patients with HF as it has little to no effect on mineralocorticoid receptors [69]. More research is required to clarify the safety and efficacy of oral steroids as an adjuvant to diuretic therapy in patients with HF.

## Salt Supplements and Hypertonic Saline

The ESC HF guidelines recommend limiting daily salt intake to < 5 g [70]. However, salt restriction is associated with greater neurohormonal activation, [71] and an observational study suggested it may be associated with an increased risk of HF hospitalisation [72]. An RCT comparing salt-restricted diet (< 1.5 g per day) to standard care in ambulatory out-patients with HF found no difference in morbidity or mortality [73]. An RCT of salt and water restriction in patients hospitalised with worsening HF suggested that it did not improve control of congestion but increased thirst. [74].

Meta-analysis of a series of small trials suggests that infusing hypertonic saline (HS) with high-dose IV furosemide increases diuresis, shortens hospital stay, and reduces HF re-admissions [75]. The mechanism of benefit of HS is not well understood: increased renal blood flow, [76] increased cardiac output, [77, 78] and reduced neurohormonal activation [79] are all putative mechanisms. The diuretic effect may simply be due to increased natriuresis in response to an increase in serum sodium concentration. [80].

In the largest RCT of hypertonic saline to date, 1927 patients admitted to the hospital with HF and low urine output (< 0.8 L per day) despite high dose oral loop diuretic were randomised to hypertonic saline (1.4–4.6% depending on serum sodium concentrations) plus 250 mg IV furosemide BD or 250 mg IV furosemide BD alone. At discharge, patients randomised to the HS arm were encouraged to take a liberal salt diet (120 mmol per day), and those in the loop diuretic-only arm were encouraged to take a restricted salt diet (80 mmol per day). The primary endpoint was death or HF hospitalisation, and secondary endpoints included daily diuresis, as well as change in body weight and renal function from randomisation to discharge. HS was associated with shorter length of stay (3.5 vs. 5.5 days), greater diuresis (2150 mL vs. 1675 mL per day), greater reduction in body weight from admission to discharge (9.5 kg vs. 7.9 kg), and an improvement in renal function. Median follow-up was 57 months during which time 12.9% died and 18.5% were re-admitted for those assigned to HS, compared to 23.8% and 34.2% in the control group [11•]. Concerns around data veracity have limited adoption in guidelines. [81, 82].

Regardless of the supporting data, widespread use of HS in patients admitted to hospital with HF is logistically difficult. HS can cause phlebitis, and a rapid increase in serum sodium concentration can cause osmotic demyelination syndrome leading to irreversible neurological damage. As a result, HS infusions are often given via a central vein and under close monitoring in high-dependency or intensive care units [83]. Although some studies suggest that peripheral administration of HS is safe, [84] it is not a routine practice.

Oral sodium chloride (Slow-Sodium®) in doses of up to 12 g per day (20 tablets) may be a pragmatic alternative to IV HS. The OSPREY trial included 65 patients admitted to the hospital with HF (mean age 70; mean LVEF 45%; median NTproBNP 4040 ng/L; mean eGFR 39 ml/min/1.73 m^2^) all of whom were taking high-dose oral loop diuretic prior to hospitalisation (mean furosemide-equivalent of 770 mg per day). Patients were randomised to 6 g of oral salt per day or placebo for 4 days. Oral salt had no effect on change in body weight or renal function (primary endpoints). The median dose of diuretic was 460 mg per day in the oral salt arm and 405 mg per day in the placebo arm, and total urine output over 4 days was numerically (but not statistically) greater in the oral salt arm (10.0 L vs. 9.4 L; *P* = 0.61). Oral salt was associated with a smaller reduction in serum sodium concentration (− 0.03 mEq/L vs. − 2.60 mEq/L; *P* < 0.001) and a smaller increase in serum urea (3.1 mEq/L vs. 11.0 mEq/L; *P* = 0.025) compared to placebo. Oral salt was well tolerated with no serious adverse events related to treatment reported. [12].

It may be that the dose of oral salt given was too low, or that gastrointestinal absorption of salt was impaired by gut wall oedema. Although the dose and route of administration were sufficient to affect serum sodium concentration, this had no effect on diuresis. While HS or oral salt supplements might be beneficial, the former is logistically challenging, and robust evidence for the latter is lacking.

## Directions for Research

**Fig. 3:**
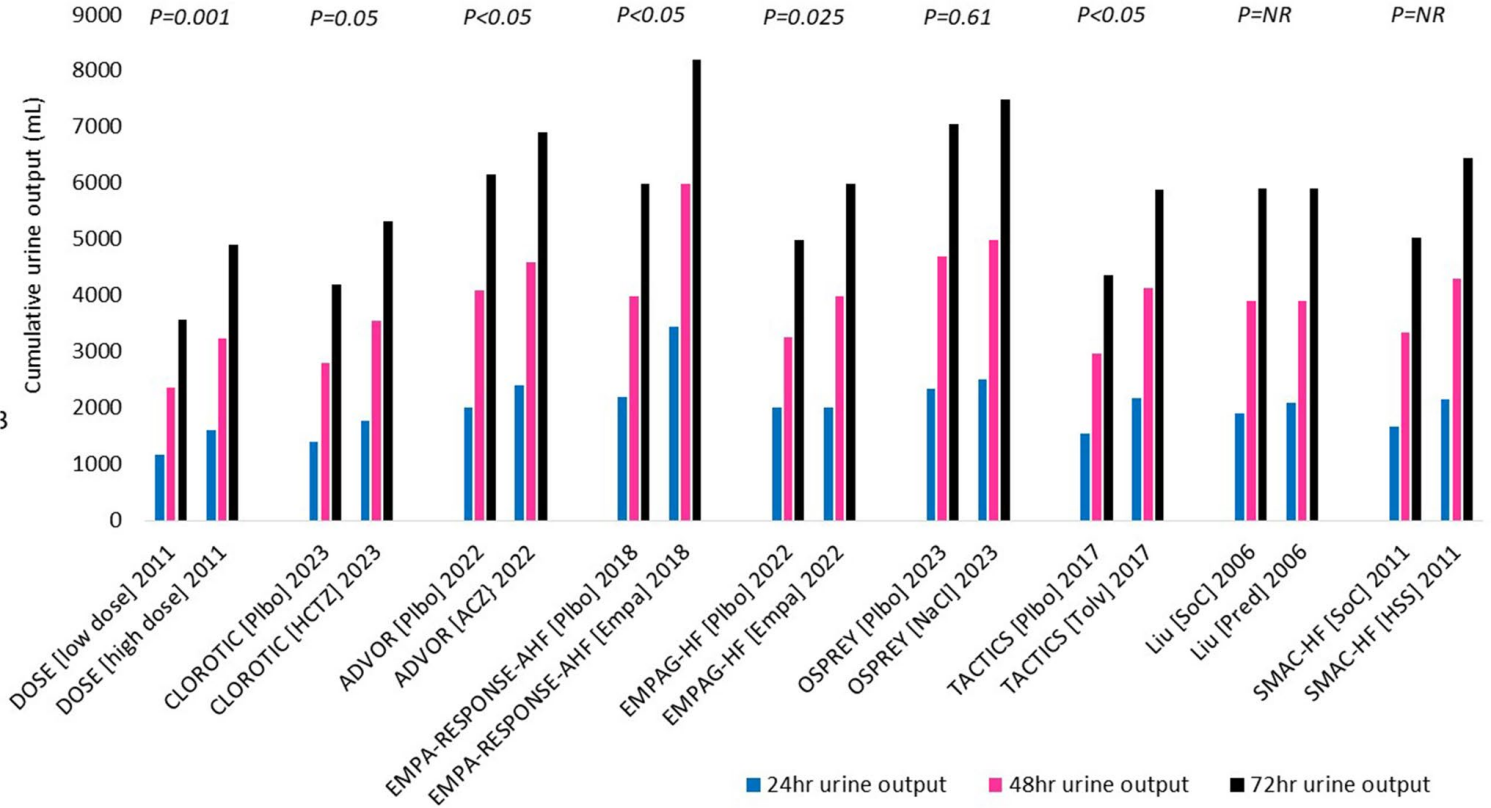
Cumulative urine output in RCTs of combination diuretic therapy. Urine output was reported at 24 h in the CLOROTIC and SMAC-HF trials and was used to estimate urine output at 48 and 72 h; urine output was reported at 72 h in the DOSE and TACTICS trials and used to estimate urine output at 24 and 48 h. Urine output from the ADVOR, EMPA-RESPONSE-AHF, EMPAG-HF, OSPREY, and Liu et al. trials was estimated from the figures. Data collection on urine output stopped after 48 h in the ADVOR trial; 72 h urine output is estimated from values at 24 and 48 h. Abbreviations: plbo, placebo; HCTZ, hydrochlorothiazide; ACZ, acetazolamide; Empa, empagliflozin; NaCl, slow sodium; Tolv, tolvaptan; Pred, prednisolone; HSS, hypertonic saline solution

Almost all trials of combination diuretic therapy to date have been head-to-head comparisons. These suggest that any combination might enhance a furosemide-induced diuresis (Fig. 3). Comparisons between the trials are nearly impossible due to the heterogeneity in loop diuretic dosing, administration, and reporting; duration of the intervention(s); and primary and secondary endpoints (Table 4).

**Table 4:** Differences in loop diuretic dose, treatment duration, and endpoint measurement

Trial (date)	Groups	Daily loop diuretic dose (how reported)	Duration of treatment	Primary endpoint (time point)	Other diuretic endpoints (time point)
CLOROTIC (2022) [7]	HCTZ (variable doses) vs. placebo	80 mg (calculated from cumulative dose)	5 days	Change in body weight (day 3) Change in patient-reported breathlessness on VAS (day 3)	Diuresis (day 1) Weight loss per 40 mg of FE (days 3 and 4)
ADVOR (2022) [7]	ACZ (500 mg OD IV) vs. placebo	120 mg (reported in supplement)	2 days	Successful decongestion (day 3)	Cumulative diuresis (days 1 and 2) Cumulative natriuresis (days 1 and 2)
EMPA-RESPONSE-AHF (2018) [8]	Empagliflozin (25 mg) vs. placebo	80 mg (calculated from cumulative dose)	30 days	Change in patient-reported breathlessness on VAS (day 4) Weight loss per 40 mg of FE (day 4) Duration of hospitalisation Change in NTproBNP (day 4)	Cumulative urine output (days 1–4) Cumulative fluid balance (days 1–4) Weight loss (day 4)
EMPAG-HF (2022) [31]	Empagliflozin (10 mg) vs. placebo	70 mg (calculated from cumulative dose)	5 days	Total urine output (day 5)	Weight loss (day 5)
TACTICS (2017) [36]	Tolvaptan (30 mg) vs. placebo	71 mg (reported in main text)	2 days	Moderate improvement in breathlessness on Likert scale (day 1)	Weight loss (days 1–3) Cumulative fluid balance (days 1–3) Successful decongestion (days 1–3)
SECRET of CHF (2017) [9]	Tolvaptan (30 mg) vs. placebo	160 mg (derived from the figure)	7 days	Change in patient-reported breathlessness on the Likert scale (day 1)	Weight loss (days 1–3)

HCTZ, hydrochlorothiazide; VAS, visual analogue scale; FE, furosemide equivalents; ACZ, acetazolamide; OD, once daily; IV, intravenous; NTproBNP, N-terminal pro-B-type natriuretic peptide.

Loop diuretic monotherapy has been the foundation of diuretic therapy for decades. DOSE and PUSH-AHF have demonstrated that high-dose furosemide is safe and more effective than lower doses. While there are several possible adjunctive therapies, most are reserved for patients with diuretic resistance in clinical practice, and none has robust data to support their use. Trials of IV furosemide plus adjunctive therapy compared to high-dose IV furosemide alone, initiated early after admission for patients with evidence of gross water retention and congestion, are needed.

However, such a trial will be difficult to design and perform.

- There is wide variation in IV furosemide dosing and little agreement on whether continuous or bolus dosing should be standard practice.
- Duration of treatment is uncertain. Almost all trials of acute diuretic strategies (apart from those using SGLT2I) have treated patients for only 2–5 days. Unsurprisingly, none has shown an effect on medium- to long-term outcomes.
- There are no data to guide recommendations on the optimal dose of oral loop diuretic to prescribe at the point of discharge, although there is a general consensus that it should be greater than the dose the patient was taking on admission to the hospital [85]. Better in-patient diuresis might lead to under-dosing at discharge.
- Guidelines recommend that patients should be euvolaemic at discharge, [14] and the rationale for oral diuretic on discharge is to prevent recurrence of congestion. However, many patients leave the hospital with residual signs of congestion, [86] and those who do are at greater risk of adverse outcomes. [87, 88] Sub-clinical venous congestion (detected on ultrasound) is common in patients with HF and is associated with a higher risk of adverse outcome [89]. Inadequate dosing of oral diuretic at discharge may lead to worsening symptoms of HF and, potentially, re-admission.
- Up to 25% of patients admitted to the hospital with HF who survive to discharge will be readmitted within 30 days, the majority due to HF, renal dysfunction, or respiratory tract infection [90]. Pulmonary congestion can increase the risk of lower respiratory tract infection, and treatment with diuretic can reduce the risk of LRTI in patients with HF. [91, 92] Maintaining adjunctive therapy to diuretic therapy on discharge, at least for a few weeks, might reduce the risk of worsening HF symptoms, readmission, and death.

There is no agreed core outcome set (COS) for patients admitted to the hospital with HF. By contrast, there is a well-defined COS for research and clinical practice in out-patients with chronic HF, which includes symptoms, quality of life, exercise capacity, hospitalisations, and mortality. [93, 94

Achieving decongestion during admission and being “alive and well” at a specific time point after discharge are important outcomes for patients and clinicians. This may be achievable in a large pragmatic trial of adjuncts to diuretic therapy using a combination of established diuresis and decongestion endpoints at the point of discharge and a combination of hospitalisation and mortality endpoints and QoL measured by the KCCQ.

## Summary and Conclusion

There are several interventions that might be adjuncts to loop diuretic therapy. However, there is little agreement on how loop diuretic should be used in patients with severe fluid retention, let alone which adjunct to use and when to use it. Until trials are designed that compare different types of combination therapy with high-dose IV loop diuretic in the acute phase followed by effective maintenance therapy post-discharge, the evidence for combination diuretic therapy will remain flimsy.
